# Sleep insufficiency and incident diabetes mellitus among indigenous and minority populations in Greece

**DOI:** 10.5935/1984-0063.20200081

**Published:** 2021

**Authors:** Anestis Matziridis, Dimitrios Tsiptsios, Apostolos Manolis, Andreas Ouranidis, Andreas S Triantafyllis, Konstantinos Tsamakis, Aspasia Serdari, Eleni Leontidou, Aikaterini Terzoudi, Elena Dragioti, Paschalis Steiropoulos, Gregory Tripsianis

**Affiliations:** 1 Democritus University of Thrace, Laboratory of Medical Statistics - Alexandroupolis - Thrace - Greece.; 2 South Tyneside & Sunderland NHS Foundation Trust, Department of Clinical Neurophysiology - Sunderland - Tyne & Wear - United Kingdom.; 3 Aristotle University of Thessaloniki, Department of Chemical Engineering - Thessaloniki - Central Macedonia - Greece.; 4 Askepeion Hospital, Department of Cardiology - Athens Greece.; 5 King’s College, Institute of Psychiatry, Psychology and Neuroscience - London - United Kingdom.; 6 Democritus University of Thrace, Department of Child and Adolescent Psychiatry - Alexandroupolis - Thrace - Greece.; 7 Democritus University of Thrace, Neurology Department - Alexandroupolis - Thrace - Greece.; 8 Linköping University, Department of Health, Medicine and Caring Sciences - Linköping - Linköping - Sweden.; 9 Democritus University of Thrace, Department of Pneumonology - Alexandroupolis - Thrace - Greece.

**Keywords:** Sleep Duration, Diabetes Mellitus, Sleep Quality, Insomnia

## Abstract

**Objective:**

To investigate the potential association between sleep pathology and diabetes mellitus (DM) using self-reported questionnaires.

**Material and Methods:**

957 adults aged between 19 and 86 years old were enrolled in this cross-sectional study. Multistage stratified cluster sampling was used and subjects were classified into three groups [short (<6h), normal (6-8h) and long (>8h) sleep duration]. Individuals were classified as diabetics if they responded positively to the questions: “Have you ever been told that you are diabetic or have high blood sugar by a health professional?” or “Are you on antidiabetic medication?”. Sleep quality, utilizing Epworth sleepiness scale, Athens insomnia scale, Pittsburgh sleep quality index and Berlin questionnaire, was also examined.

**Results:**

DM prevalence was higher among expatriated and Muslim Greeks (23.1% and 18.7%, respectively) compared to indigenous Greek Christians (4.4%). DM prevalence was significantly associated with short sleep duration (aOR=2.82, p<0.001), excessive daytime sleepiness (aOR=2.09, p=0.019) and poor sleep quality (aOR=2.56, p<0.001), while its relation with insomnia (aOR=1.63, p=0.065) and risk for obstructive sleep apnea (aOR=1.53, p=0.080) were of marginal statistical significance.

**Conclusion:**

This study indicates an association between sleep quantity, quality and DM and supports early pharmacological and cognitive behavioral interventions on sleep disturbances in order to reduce the burden of DM with increased focus on minority population needs.

## INTRODUCTION

Sleep is perceived as a complex dynamic process regulated by homeostatic and circadian effects. The iterated architecture is orchestrated by discrete hormonal changes, pertaining to significant glucose level and metabolism responses^1^. Habitual short sleep duration is linked to various morbidities, i.e., cardiometabolic abnormalities, stress, and obesity^2^, thus posing an unmet societal challenge^3^ prioritized for urgent health action plans^4^.

Diabetes mellitus (DM) is another prevalent lifelong disease characterized by elevated systemic glucose levels, owing to autoimmune destruction of β cells of the endocrine pancreas in case of type 1 DM (T1DM) or metabolic dysfunction and inflammation leading to insufficient pancreatic production of insulin and inefficient cellular absorption of glucose in case of type 2 DM (T2DM)^5^. According to WHO report of 2020, 422 million people worldwide currently suffer from DM, particularly in low and middle-income regions^6^.

Based on existing literature sleep disorders are more prevalent amongst subjects suffering from DM when compared to healthy populations^7^. In case of DM sleep disorders may be attributed to variable factors, such as peripheral neuropathy, restless limb syndrome, abrupt glucose level changes fostering either hyper - or hypoglycemic disorders, sleep apnea, and nocturia^8^. Conversely, diabetic individuals present increased risk of depression and co-morbidity development, which affect critically the quality and duration of their sleep. Neurotransmitter, neurobehavioral and autonomic impact, adversely affect endocrine functions fostering sleep disorders^9^. Consequently, it is considered important to simultaneously study variables related to DM and sleep disturbance.

Since there exists no golden standard of human habitual sleep duration measurement, various methodologies, i.e., sleep diary, actigraphy and survey of modal or average sleep calculation have been implemented for such evaluations. Among them, self-reported questionnaires consist a valuable enabling tool to assess sleep disturbances^10^, aid insomnia diagnosis, and investigate various parameters, thus providing researchers with the opportunity to extrapolate the results in large population samples^11,12^, as in case of DM patient cohorts^13,14^.

In recently published cross-sectional studies, our research team utilizing self-reported questionnaires exhibited that short sleep duration and poor sleep quality are associated with increased prevalence of anxiety^15^ and depression^16^ in the primary care setting in Greece. In this paper, we aim to reveal possible correlations between sleep quantity and quality and DM, studying a regional representative Greek population cohort and taking into account several socio-demographic characteristics, lifestyle habits and health related characteristics of the participants.

## MATERIAL AND METHODS

### Study sample and research design

The study population in this cross-sectional study consisted of 957 participants, 439 (45.9%) males and 518 (54.1%) females, with a mean age of 49.62±14.79 years (range, 19-86 years; median age, 50 years). The research design of this study is reported in Serdari et al. (2020)^15^.

### Ethics

All procedures performed in the study were in accordance with the ethical standards of the Democritus University Ethics Committee, which approved its conduct, and with the standards of the Helsinki declaration (1964) and its later amendments. Informed consent was obtained from all participants of the study.

### Covariates

A structured questionnaire was used to collect: a. standard socio-demographic characteristics (gender, age, place of residence, education level, marital, cultural, and employment status); b. lifestyle and dietary habits (smoking status, alcohol consumption, daily coffee consumption, caffeine consumption in the evening, adherence to the Mediterranean diet^17^, time watching TV or using a computer before bedtime, physical activity, and nap during the day); and c. health related characteristics (subjective general health status, body mass index, chronic disease morbidity, depression^18^ and anxiety symptoms^19^, and use of sleep medication) of the participants (Appendix).

### Measures of sleep

Participants provided information on their nighttime sleep by answering the following sleep questions of the questionnaire: “At what time do you normally go to bed?”, “At what time do you normally get up?” and “On average, how many hours do you sleep per day?” (Appendix). Responses were obtained for an average weekday and weekend day over the previous month. Time in bed was calculated as the difference between bedtime and rise time. As a proxy of the overall time in bed or sleep duration on a weekly basis, weighted mean measures were calculated using the following formulas: weighted time in bed = 5/7*(time in bed on a weekday) + 2/7*(time in bed on a weekend day) and weighted sleep duration = 5/7*(sleep duration on a weekday) + 2/7*(sleep duration on a weekend day). Sleep efficiency refers to the percentage of time a person sleeps in relation to the amount of time a person spends in bed and was calculated as the ratio of sleep duration and time in bed X 100.

Participants were then classified into the following three sleep categories according to their sleep duration: short (<6 hours), normal (6-8 hours) and long sleep duration (>8 hours). Finally, in order to assess basic difficulties on sleep patterns over the previous month, participants were asked about the frequency of difficulty falling asleep, maintaining asleep and early morning awakening.

### Assessment of sleep disturbances

In order to assess daytime sleepiness, insomnia, sleep quality and risk of obstructive sleep apnea (OSA), the participants filled in the following standardized scales in Greek version, Epworth sleepiness scale (ESS)^20^, Athens insomnia scale (AIS)^21^, Pittsburgh sleep quality index (PSQI)^22^, and Berlin questionnaire (BQ)^23^, respectively (Appendix).

### Definition of DM

Participants were classified as diabetics if they responded positively to the questions “Have you ever been told that you are diabetic or have high blood sugar by a health professional?” or “Are you on antidiabetic medication?”^13,14^.

### Statistical analysis

Statistical analysis of the data was performed using IBM Statistical Package for the Social Sciences (SPSS), version 19.0 (IBM Corp., Armonk, NY, USA). The normality of quantitative variables was tested with Kolmogorov-Smirnov test. Quantitative variables were expressed as mean ± standard deviation (SD) and qualitative variables were expressed as absolute and relative (%) frequencies. In particular, mean estimated time of sleep characteristics (i.e., bedtime, rise time, time in bed, and sleep duration) were expressed as HH:MM. We conducted the following analyses: (i) in the univariate analysis, the association of diabetes with subjects’ characteristics, sleep characteristics and sleep disorders was assessed using the chi-square test and Student’s t-test; (ii) multivariate stepwise logistic regression analysis was used to explore the independent risk factors for diabetes, controlling for all subjects’ characteristics; (iii) for the evaluation of the effect of sleep duration and sleep disorders on the prevalence of diabetes, two different logistic regression models were constructed: model 1 (crude, unadjusted) and model 2 (adjusted for subjects’ socio-demographic, lifestyle habits and health related characteristics). Odds ratios (OR) with their 95% confidence intervals (CI) were estimated as the measure of the above associations.

Receiver operating characteristic (ROC) analysis was used to provide the ability of sleep duration to classify subjects with diabetes. The area under the ROC curve (AUC), sensitivity and specificity were estimated. The optimal cutoff value of the sleep duration that differentiates diabetics from non-diabetics was derived according to Youden index. All tests were two tailed and statistical significance was considered for *p*-values<0.05.

## RESULTS

### Subjects’ characteristics

91 individuals (9.5%) were classified as diabetics. The prevalence of DM in relation to participants’ socio-demographic, lifestyle and health related characteristics is summarized in Tables 1 and 2.

**Table 1.** Prevalence of DM in relation to subjects’ demographic characteristics.

		DM		
	Total sample	Frequency	Proportion (%)	p-value
Gender				<0.001
Females	518 (54.1)	28	5.4	
Males	439 (45.9)	63	14.4	
Age				<0.001
≤40 years	273 (28.5)	16	5.9	
41 – 60 years	444 (46.4)	20	4.5	
>60 years	240 (25.1)	55	22.9	
Marital status				<0.001
Married	645 (67.4)	68	10.5	
Single	196 (20.5)	4	2.0	
Divorced	36 (3.8)	0	0.0	
Widowed	80 (8.4)	19	23.8	
Cultural status				<0.001
Greek Christians	632 (66.1)	28	4.4	
Greek Muslims	273 (28.5)	51	18.7	
Expatriated Greeks	52 (5.4)	12	23.1	
Place of residence				<0.001
Urban	416 (43.5)	16	3.8	
Rural	541 (56.5)	75	13.9	
Education level				<0.001
Low	313 (32.7)	67	21.4	
Medium	340 (35.5)	20	5.9	
High	304 (31.8)	4	1.3	
Working Status				0.129
Employed	872 (91.1)	79	9.1	
Unemployed	85 (8.9)	12	14.1	
Financial status (n=812)				<0.001
Low	476 (49.7)	75	15.8	
Medium	200 (20.9)	12	6.0	
High	136 (14.2)	0	0.0	

**Table 2.** Prevalence of DM in relation to subjects’ lifestyle habits and health related characteristics.

		DM		
	Total sample	Frequency	Proportion (%)	p-value
Smoking ever				0.001
Never smoked	369 (38.6)	19	5.1	
Ex-smoker	255 (26.6)	35	13.7	
Current smoker	333 (34.8)	37	11.1	
Alcohol consumption				<0.001
Never	488 (51.0)	70	14.3	
Occasionally or daily	469 (49.0)	21	4.5	
Coffee consumption				0.003
None	84 (8.8)	8	9.5	
1 - 2 cups/day	564 (58.9)	51	9.0	
3 - 4 cups/day	260 (27.2)	20	7.7	
> 4 cups/day	49 (5.1)	12	24.5	
Caffeine consumption in the evening (>6 p.m.)				0.146
No	415 (43.4)	46	11.1	
Yes	542 (56.6)	45	8.3	
Number of meals				0.002
1 meal	20 (2.1)	0	0.0	
2 meals	353 (36.9)	20	5.7	
>2 meals	584 (61.0)	71	12.2	
Adherence to MED diet				<0.001
Low	743 (77.6)	84	11.3	
High	214 (22.4)	7	3.3	
Time watching TV or using a computer before bedtime				0.304
<1 hour	120 (12.5)	8	6.7	
1 - 2 hours	326 (34.1)	28	8.6	
>2 hours	511 (53.4)	55	10.8	
Physical activity				<0.001
Low	805 (84.1)	91	11.3	
High	152 (15.9)	0	0.0	
Nap during the day				0.008
No	721 (75.3)	79	11.0	
Yes	236 (24.7)	12	5.1	
Subjective health status				<0.001
Bad	220 (23.0)	59	26.8	
Good	737 (77.0)	32	4.3	
BMI status				<0.001
Normal	328 (34.3)	12	3.7	
Overweight	272 (28.4)	27	9.9	
Obese	357 (37.3)	52	14.6	
Anxiety symptoms				0.015
No	635 (66.4)	50	7.9	
Yes	322 (33.6)	41	12.7	
Depression symptoms				<0.001
No	685 (71.6)	40	5.8	
Yes	272 (28.4)	51	18.8	
Use of sleep medication				0.453
No	891 (93.1)	83	9.3	
Yes	66 (6.9)	8	12.1	

### DM and sleep habits

The association of DM with subjects’ sleep characteristics is shown in Table 3. The weighted weekly time in bed and sleep duration were calculated and compared between the two groups; it was noted that, although diabetics used to spent 23 min longer time in bed (*p*=0.001), they reported a 26 min shorter sleep duration (*p*=0.005) and lower sleep efficiency (*p*<0.001) compared to non-diabetics. All the above relations between DM and sleep characteristics remained unchanged among females and males. In particular, females with DM used to sleep 44 min less than females without DM (*p*<0.001) and males with DM used to sleep 27 min less than males without DM (*p*<0.001). Among subjects with DM, all three sleep characteristics were similar between males and females (time in bed: *p*=0.882; sleep duration: *p*=0.356; sleep proportion: *p*=0.171).

**Table 3.** Association of diabetes with sleep characteristics.

	Total sample	Diabetes		Difference*	p-value
		No	Yes		
Weekdays sleep habits					
Bedtime	11:29 (1:05)	11:34 (1:05)	10:44 (0:42)	-50 (4.3)	<0.001
Rise time	6:53 (1:01)	6:55 (1:02)	6:34 (0:47)	-21 (4.0)	0.002
Time in bed	7:24 (1:05)	7:21 (1:05)	7:50 (0:58)	29 (4.3)	<0.001
Sleep duration	6:19 (1:11)	6:20 (1:09)	6:02 (1:25)	-18 (4.7)	0.046
Sleep efficiency (%)	86 (12)	87 (11)	77 (12)	-10 (1.2)	<0.001
Weekends sleep habits					
Bedtime	11:55 (1:19)	12:02 (1:18)	10:51 (0:43)	-71 (5.0)	<0.001
Rise time	7:46 (1:32)	7:52 (1:34)	6:47 (0:47)	-65 (5.8)	<0.001
Time in bed	7:50 (1:00)	7:50 (1:01)	7:56 (0:52)	6 (4.0)	0.274
Sleep duration	6:45 (1:16)	6:49 (1:13)	6:05 (1:24)	-44 (5.0)	<0.001
Sleep efficiency (%)	86 (12)	87 (11)	76 (12)	-11 (1.2)	<0.001
Weekly sleep habits					
Total sample					
Time in bed	7:32 (1:00)	7:29 (1:00)	7:52 (0:56)	23 (4.0)	0.001
Sleep duration	6:26 (1:10)	6:29 (1:08)	6:03 (1:25)	-26 (4.7)	0.005
Sleep efficiency (%)	86 (12)	87 (11)	77 (12)	-10 (1.2)	<0.001
Females					
Time in bed	7:36 (0:59)	7:35 (1:00)	7:54 (0:57)	19 (6.8)	0.109
Sleep duration	6:30 (1:10)	6:32 (1:08)	5:48 (1:24)	-44 (7.9)	0.001
Sleep efficiency (%)	86 (12)	87 (12)	74 (12)	-13(2.2)	<0.001
Males					
Time in bed	7:26 (1:00)	7:23 (1:00)	7:51 (0:53)	28 (4.7)	0.001
Sleep duration	6:22 (1:10)	6:36 (1:06)	6:09 (1:25)	-27 (5.4)	0.191
Sleep efficiency (%)	86 (11)	87 (10)	78 (12)	-9 (1.4)	<0.001

* mean difference (S.E.) between subjects with and without DM, expressed as minutes (bedtime, rise time, time in bed, and sleep duration) and as percentages (sleep efficiency).

In the sequence, according to the self-reported sleep duration, participants were categorized into three groups: short (<6h), normal (6-8h) and long (>8h) sleep duration. The association between DM and sleep duration, which was considered as a categorical variable (Table 4), revealed that DM was significantly more frequent (*p*<0.001) in subjects with short (16.7%) compared to those with normal (7.2%) and long (8.8%) sleep duration. The association of DM with sleep duration had the same pattern in both genders (*p*=0.033 for females; *p*=0.040 for males). In particular, logistic regression analysis revealed that in subjects with short sleep duration there were more than 2.5-times higher odds for DM compared to subjects with normal sleep duration (OR=2.59, *p*<0.001). A 2.96-fold (*p*=0.012) and a 2.07-fold (*p*=0.015) increase in odds of DM was associated with short sleep duration in females and males, respectively.

**Table 4.** Association of sleep duration with DM in relation to gender using logistic regression models.

			Model 1		Model 2	
	Diabetes n (%)	p-value	cOR (95% CI)	p-value	aOR (95% CI)	p-value
Total sample						
Sleep duration		<0.001				
Short	35 (16.7)		2.59 (1.61-4.18)	<0.001	2.82 (1.70-4.70)	<0.001
Normal	44 (7.2)		Ref.		Ref.	
Long	12 (8.8)		1.25 (0.64-2.44)	0.513	1.27 (0.65-2.50)	0.488
Females						
Sleep duration		0.033				
Short	10 (10.9)		2.96 (1.27-6.91)	0.012	2.51 (0.99-6.49)	0.033
Normal	14 (4.0)		Ref.		Ref.	
Long	4 (5.6)		1.43 (0.46-4.47)	0.540	1.26 (0.35-4.50)	0.719
Males						
Sleep duration						
Short	25 (21.4)	0.040	2.07 (1.15-3.70)	0.015	2.53 (1.31-4.88)	0.006
Normal	30 (11.6)		Ref.		Ref.	
Long	8 (12.5)		1.09 (0.47-2.50)	0.847	1.01 (0.43-2.36)	0.987

cOR = crude odds ratio; aOR = Adjusted odds ratio; CI = confidence interval; model 1 = Crude, unadjusted; model 2 = Adjusted for socio-demographic characteristics, lifestyle habits (smoking status, alcohol consumption, daily coffee consumption, caffeine consumption in the evening, adherence to the Mediterranean diet, time watching TV or using a computer before bedtime, physical activity, nap during the day) and health related characteristics (subjective general health status, BMI, chronic disease morbidity, anxiety, depression, and use of sleep medication).

### Independent effect of DM on sleep habits

Two separate multivariate logistic regression models, controlling for the effect of all subjects’ socio-demographic, lifestyle and health related characteristics, were constructed in order to assess the independent effect of sleep duration on the prevalence of DM. When sleep duration was entered in the model as a continuous variable, it remained a statistically significant independent determinant of increased odds for diabetes (*p*=0.002); in particular, shorter sleep duration by one hour was associated with a 41%-increase in the odds for DM (aOR=1.41, 95% CI=1.13-1.76).

When sleep duration was entered in the multivariate logistic regression model as a categorical variable, the inverse relationship between DM and sleep duration persisted even after the adjustment for all potential confounders. In particular, the odds of DM were almost 3 times higher for subjects sleeping less than 6 hours (aOR=2.82, *p*<0.001) compared to those with normal sleep duration; the respective odds for DM were similar in the two genders (aOR=2.51, *p*=0.033 in females; aOR=2.53, *p*=0.006 in males). Sleeping longer than 8 hours showed no significant effect on the development of DM (Table 4).

Moreover, the area under the ROC curve (AUC) showed that sleep duration has a significant ability to discriminate subjects with DM (AUC=0.634, 95% CI=0.564-0.702, *p*=0.035). The optimal cut-off point of sleep duration of 5:33 hours, which was determined to classify subjects with DM, yielded high sensitivity of 60.4% and specificity of 77.4%. Sleep duration showed significant discrimination ability in both genders, although its performance was superior among females (females: AUC=0.729, 95% CI=0.617-0.842, *p*<0.001, cut-off ≤6:04hours, sensitivity=85.7%, specificity=66.6%; males: AUC=0.579, 95% CI=0.493-0.664, *p*=0.045, cut-off ≤5:38hours, sensitivity=55.5%, specificity=73.5%).

### DM and sleep disorders

According to the Greek versions of ESS, AIS, PSQI and BQ the prevalence of daytime sleepiness was 8.7% (83 subjects), insomnia 18.0% (172 subjects), poor sleep quality 38.5% (368 subjects) and high risk of obstructive sleep apnea 36.4% (348 subjects). The internal consistency of all four questionnaires was very high (Cronbach α coefficient was ranged from 0.74 to 0.88). The development of DM in relation to sleep disorders is shown in Table 5. Univariate statistical analysis showed that DM was more frequent in subjects with excessive daytime sleepiness (20.5% vs 8.5%, *p*<0.001), insomnia (14.0% vs 8.5%, *p*=0.028), poor sleep quality (14.4% vs 6.5%, *p*<0.001) and high risk for OSA (12.6% vs 7.7%, *p*=0.012). In multivariate logistic regression analysis controlling for all subjects’ characteristics, the odds of DM remained significantly associated with excessive daytime sleepiness (aOR=2.09, *p*=0.019) and poor sleep quality (aOR=2.56, *p*<0.001), while its relation with insomnia (aOR=1.63, *p*=0.065) and the risk for OSA (aOR=1.53, *p*=0.080) were of marginal statistical significance.

**Table 5.** Association of sleep questionnaires and sleep difficulties with DM using logistic regression models.

	Diabetes n (%)	p-value	cOR (95% CI)	Model 1	aOR (95% CI)	Model 2
				p-value		p-value
**Sleep questionnaires**						
ESS		<0.001				
Normal day sleepiness	74 (8.5)		Ref.		Ref.	
Excessive day sleepiness	17 (20.5)		2.79 (1.55-4.99)	<0.001	2.09 (1.13-3.89)	0.019
AIS		0.028				
Non-insomniac	67 (8.5)		Ref.		Ref.	
Insomniac	24 (14.0)		1.74 (1.06-2.86)	0.028	1.63 (0.97-2.73)	0.065
PSQI		<0.001				
Good quality	38 (6.5)		Ref.		Ref.	
Bad quality	53 (14.4)		2.44 (1.57-3.78)	<0.001	2.56 (1.61-4.06)	<0.001
BQ		0.012				
Low risk	47 (7.7)		Ref.		Ref.	
High risk	44 (12.6)		1.73 (1.12-2.67)	0.012	1.53 (0.95-2.47)	0.080
**Sleep difficulties **						
Delay in falling asleep		0.938				
Less than once a week	57 (9.5)		Ref.		Ref.	
At least once a week	34 (9.6)		1.02 (0.65-1.59)	0.938	1.22 (0.76-1.95)	0.404
Inability to stay asleep		<0.001				
Less than once a week	16 (4.4)		Ref.		Ref.	
At least once a week	75 (12.6)		3.12 (1.79-5.44)	<0.001	1.25 (1.24-4.08)	0.007
Waking-up too early		0.004				
Less than once a week	40 (7.2)		Ref.		Ref.	
At least once a week	51 (12.8)		1.89 (1.22-2.92)	0.004	1.88 (1.18-3.01)	0.008

ESS, Epworth Sleepiness Scale; AIS, Athens Insomnia Scale; PSQI, Pittsburgh Sleep Quality Index; BQ, Berlin Questionnaire; cOR, crude Odds Ratio; aOR, adjusted Odds Ratio; CI, confidence interval; model 1, crude, unadjusted; model 2, adjusted for all socio-demographic characteristics, lifestyle habits and health related characteristics.

Regarding to the basic difficulties of sleep patterns, significant increased odds of DM were found among subjects who reported difficulties in maintaining sleep (aOR=2.25, *p*=0.007) and early morning awakenings (aOR=1.88, *p*=0.008), but not difficulties initiating sleep (aOR=1.22, *p*=0.404).

## DISCUSSION

A cross-sectional study utilizing a population-based sample from the rural region of Thrace in northeastern Greece was conducted in order to evaluate the potential associations of adults’ sleep habits and disturbances with DM in the primary care setting. It was revealed that DM was more prominent among minority groups. Moreover, DM was not only associated with shorter sleep duration and reduced sleep efficiency, but also with poor sleep quality.

The high evidenced prevalence of DM, i.e., 9.5% of the sample population studied, is consistent with the International Diabetes Federation, according to which, the median global prevalence of DM is projected from 382 million (8.3%) in 2013 to reach 592 million (10.1%) in 2035^24^. Moreover, the prevalence of DM was noted higher among Expatriated and Muslim Greeks displaying proportions of 23.1% and 18.7%, respectively, against the Greek Christians group 4.4% (Table 1). This finding supports the hypothesis that minority groups follow inconsistent, unhealthy living and feed habits. The increased prevalence of DM in minority groups has been demonstrated in several studies^25,26^. Furthermore, rural citizens carry the disease almost fourfold compared to inhabitants of urban areas. This finding is in agreement with education level and poverty dependences, hence highly educated people presumed to live in urban areas appear privileged hence presenting less diabetic incidents. Indeed low-income populations in Western economies are found to develop DM more frequently^27^. Factors such as unhealthy feeding habits, obesity and stress contribute to higher DM frequency of unprivileged populations. The latest has been attributed to the fact that individuals of low socioeconomic status present less glycemic control prioritization, personal vulnerability and lack of easy access to health care providers^28^.

As evidenced in Table 3, diabetics spend 23 min longer time in bed (*p*=0.001) and report 26 min shorter sleep duration (*p*=0.005) and lower sleep efficiency (*p*<0.001) compared to non-diabetics. Implementing multivariate logistic regression analysis, reduced sleep duration by one hour was associated with a 41% increase in the risk for DM occurrence (aOR=1.41, 95% CI=1.13-1.76), i.e., sleep duration demonstrates a significant ability to discriminate subjects with DM. Moreover, the odds of DM were almost 3 times higher for subjects sleeping less than 6 hours (aOR=2.82, *p*<0.001) compared to those with normal sleep duration (Table 4). Our results are in keeping with a recent meta-analysis by Shan et al. (2015)^29^ that concluded that reduced sleep duration is associated with significantly increased risk of DM. In contrast, in our study a relationship between long sleep duration and DM incidence could not be identified (aOR=1.27, *p*=0.488). Von Ruesten et al. (2012)^30^ and Lin et al. (2016)^31^ could also not trace a link between long sleep duration and DM.

Certain studies evaluate duration of sleep as a three and others as four level factorial making comparison analysis hard to implement. The American Academy of Sleep Medicine and Sleep Research Society and the National Sleep Foundation advocated by the recently released relevant recommendations for adult sleep duration, according to which 7 or more hours of sleep are deemed necessary to support health^32,33^. The American Thoracic Society also reached the consensus warning that 6h or less of sleep duration is heavily associated with disease conditions, including DM^34^. For our study, the optimal cut-off point was set at 5:33hours, which was determined to classify subjects with DM, yielding high sensitivity of 60.4% and specificity of 77.4%.

With regards to sleep quality, our multivariate logistic regression analysis revealed that DM was significantly associated with excessive daytime sleepiness (aOR=2.09, *p*=0.019) and poor sleep quality (aOR=2.56, *p*<0.001), while its relation with insomnia (aOR=1.63, *p*=0.065) and the risk for OSA (aOR=1.53, *p*=0.080) were of marginal statistical significance (Table 5). Ogilvie and Patel (2018)^35^ also concluded that OSA, poor sleep quality and insomnia were more prevalent in diabetics. Concerning insomnia subtypes, significant risk of DM was found among subjects who reported difficulties maintaining sleep (aOR=2.25, *p*=0.007), and early morning awakening (aOR=1.88, *p*=0.008), but not, difficulties initiating sleep (aOR=1.22, *p*=0.404). In contrast, in a recent meta-analysis, Cappuccio et al. (2010)^36^ exhibited that both difficulties initiating and maintaining sleep are associated with increased risk of DM.

Concerning the pathophysiological link between sleep pathology and DM, it has been revealed that in case of T2DM sleep deprivation is related to decreased insulin sensitivity or insulin resistance^7,37,38^. Metabolism related hormones leptin and ghrelin secreted by the adipose tissue and stomach, might be involved in satiety signaling, hunger stimulation and implicated in DM development. Sleep laboratory studies have also shown that acute sleep deprivation decreases leptin and increases ghrelin^39^ fostering DM development. Moreover, deficient sleep contributes not only to reduced insulin release after meals, thereby maintaining glucose in the bloodstream, but also increased insulin production in an attempt to lower the elevated glucose levels attributable to increased cortisol circulating in the body following sleep loss. Furthermore, elevations in epinephrine due to increased sympathetic nervous system activity inhibit insulin release and promote glycogenolysis^5^. In case of T1DM, data that elucidate the potential causal role of sleep duration in glycemic control and vice versa are limited. Nevertheless, it has been suggested that adults and children with T1DM exhibit altered sleep architecture and reduced sleep quality due to both behavioral and physiological aspects of diabetes and its management. Apart from that, impaired glycemic control has been linked to OSA that is more prevalent in patients with T1DM. Moreover, it has been proposed that lack of the normal decline in blood pressure during sleep may be linked to short sleep duration in people T1DM^40^. Furthermore, an experimental sleep restriction study by Donga et al. (2010)^41^ showed that adults with T1DM who were limited to 4 hours of sleep exhibited lower glucose tolerance and insulin sensitivity compared to when they were provided the opportunity to obtain the recommended total sleep time. Finally, potential neuroinflammatory pathways linking T1DM and sleep pathology have also been implicated^42^.

Our analysis manifests several strengths, as it is based on data from a large representative sample of the population of the region of Thrace that provided excellent response rates to sleep quality and DM measurements. Although participation rate was not 100% our sampling scheme ensured that the sample was randomly selected and representative of the general population of this area. Limitations of this study lie in the properties of the cross-sectional study, as it is difficult to determine if there is a causal relationship between sleep pathology and DM and the recall bias of self-reported sleep duration. Nevertheless, self-report assessments of sleep have been shown to be valid measures compared with quantitative sleep assessments with actigraphy^43^. Similarly, self-reported DM could interfere in the final sample and results, as the achieved DM sample might not represent the real number of the diagnosed DM population. Even so, several studies have confirmed that the use of the datum of self-reported DM is valid^44^ as high concordance is evident between self-reported DM and medical DM record review^45^. Furthermore, due to the vast heterogeneity in the medical regimens that act through various pathophysiological pathways and taking into consideration that many patients received multiple medications from different categories it was impossible to verify any direct association between a certain regimen to sleep patterns. Finally, although the presence of well-controlled DM as indicated by normal levels of HbA1c could be associated with improved sleep quality and quantity, data on HbA1c levels are not available, thus no conclusion on this matter could be reached.

## CONCLUSION

To the best of our knowledge, this is the first cross-sectional study conducted in the primary care setting in Greece that not only presents strong evidence of an association between both sleep quantity and quality and DM but also depicts an increased prevalence of the latter in minority groups. These findings dictate the strong link of sleep disturbances with the burden of DM rendering further research on the possible interventions to improve sleep for better glucose metabolism regulation as an urgent need in this setting with special focus in minority populations.
